# Factors associated with suicidal behaviors in mainland China: a meta-analysis

**DOI:** 10.1186/1471-2458-12-524

**Published:** 2012-07-16

**Authors:** Ying Li, Yafei Li, Jia Cao

**Affiliations:** 1 Department of Social Medicine and Health Service Management, College of Preventive Medicine, Third Military Medical University, No.30 Gaotanyan Road, Shapingba District, Chongqing, People’s Republic of China; 2 Department of Epidemiology, College of Preventive Medicine, Third Military Medical University, No.30 Gaotanyan Road, Shapingba District, Chongqing, People’s Republic of China; 3 College of Preventive Medicine, Key Lab of Medical Protection for Electromagnetic Radiation, Ministry of Education of China, Third Military Medical University, No.30 Gaotanyan Road, Shapingba District, Chongqing, People’s Republic of China

**Keywords:** Risk factors, Suicide, China, Meta-analysis

## Abstract

**Background:**

Suicide is a major public health issue in China. Studies of suicide risk factors have reported both inconsistent and inconclusive results. This review aimed to determine suicide risk factors in China.

**Methods:**

Medline/PubMed, EMBASE, CNKI (China National Knowledge Infrastructure) and VIP (Chinese Journal of Science and Technology of VIP) were searched for relevant reports. Two investigators independently assessed the eligibility of identified studies and extracted data. Pooled odds ratios (and 95% confidence intervals) were calculated for each factor with Revman 5.0.

**Results:**

Forty-four studies with 192,362 subjects were included. The pooled results indicated that mood disorders and stressful life events (physical illness, suicide of relatives) increased the risk of suicide ideation among the entire population. Socio-family environment (single or remarried parent, study pressure and academic achievement) and unhealthy behaviors (smoking, alcohol drinking, and drug use) were risk factors for suicide ideation among youth. Unhealthy behaviors (smoking and alcohol drinking), mood disorders, and stressful life events (suicide of relatives) were the main risk factors for attempted suicide. Persons living in rural areas, and those with lower education, mood disorders, and/or a history of negative life events had a higher risk of completed suicide. In China, before 2000, females had a significantly higher rate of completed suicide than males, while after 2000, no significant gender difference was found.

**Conclusions:**

Socio-family environment, lifestyle, life events and psychiatric/psychological factors are associated with suicidal behaviors in China. Further case–control or cohort studies are needed to better understand suicide behaviors in China. Meanwhile, there is an urgent need for comprehensive studies of suicide interventions among high-risk populations.

## Background

Suicide is an important global public health problem. According to the World Health Organization (WHO), approximately one million people committed suicide, and 10–20 times that number attempted suicide worldwide in 2000 [1]. Globally, suicide is one of the three leading causes of death among people aged 15–34 years [2] and suicide rates have increased by 60% over the last 45 years [3]. Reducing mortality and morbidity associated with suicidal behavior is high on the WHO agenda [3].

China has long been recognized as having a high suicide rate, with 21 percent of the world’s population, but 30–40 percent of the world’s suicides [4]. Suicide patterns in China are different from those in the West. Previously in China, females had consistently higher suicide rates than males [5]. However, in recent years, this pattern has reversed. In 2008, the national male suicide rate (7.0/100,000) was higher than the national female suicide rate (6.20/100,000), as was the rural male suicide rate (4.0 vs. 3.18/100,000, respectively) and the urban male suicide rate (8.62 vs. 7.87/100,000, respectively) [5]. The suicide rate in China has substantially decreased over the last 20 years, with the 2008 national suicide rate being approximately 63 percent lower than the 1987 rate (17.65 vs. 6.60/100,00, respectively).

Despite the decreased national suicide rate, suicide is still threatening the health of youth and elderly in China. An understanding of the risk factors is imperative for effective suicide prevention. Suicide risk factors have attracted a great deal of research attention in China. Many studies have investigated the influence of demographic, socio-psychological, economic and lifestyle-related factors, and physical illness on suicidal behavior. However, the results of these studies have been inconsistent. For example, some studies found that females had a higher suicide risk than males [6,7], while others found no significant differences in risk between the sexes [8,9]. In addition, some studies [10,12] found no significant association between family type and suicide ideation, while others found that belonging to a remarried [13] or single parent [17] family increased the risk of forming suicide ideation.

This meta-analysis aimed to comprehensively examine the risk factors of completed suicide and suicidality (suicide ideation and suicide attempt) in the Chinese population. From our findings, we were able to draw a series of recommendations for future intervention programs and studies.

## Methods

### Search strategy

Electronic database and manual searches were conducted to identify published articles for review. Four databases were used to search for articles published up to January, 2011: Medline/PubMed, EMBASE, CNKI (China National Knowledge Infrastructure) and VIP (Chinese Journal of Science and Technology of VIP). The searches applied a mixture of free text and index terms. The search terms in PubMed were ("Suicide"[Mesh] AND "Risk Factors"[Mesh]) AND "China"[Mesh], ("Suicide"[Mesh] AND "China"[Mesh], (("Risk Factors"[Mesh]) AND "Suicidal Ideation"[Mesh]) AND "China"[Mesh], (("Risk Factors"[Mesh]) AND "Suicide, Attempted"[Mesh]) AND "China"[Mesh], risk factor for suicide in China, suicide in China. The results in Medline/PubMed were limited to full text, human, English and Chinese. The EMTREE search in EMBASE was 'suicide'/exp AND 'risk factor'/exp AND 'china'/exp, 'suicide'/exp AND 'china'/exp, 'risk factor'/exp AND 'suicidal ideation'/exp AND 'China'/exp, 'risk factor'/exp AND 'suicide attempt'/exp AND 'China'/exp. All results in EMBASE were limited to English, human. CNKI and VIP were used to search for articles in Chinese, and the subject words included risk factors for suicide, risk factors for suicide ideation, and risk factors for attempted suicide. We reviewed the references cited in the retrieved articles and then searched the bibliographies of these retrieved papers.

### Selection of studies

Studied design included cross-sectional, case–control, and cohort studies. Study subjects were from the general population of mainland China. Suicidal behavior outcomes included suicide ideation (thoughts of engaging in behavior intended to end one's life), suicide attempt (engagement in potentially self-injurious behavior in which there is at least some intent to die), and completed suicide (the act of intentionally ending one's life). We also included the studies that were designed with the original purpose of analyzing risk behaviors other than suicide among Chinese people, but the information on the risk factors for suicide ideation, suicide attempt or completed suicide were provided. Studies that focused on subjects from Hong Kong or Taiwan, that investigated psychopathic suicide, that repeated data reported in earlier studies, or that were systematic reviews or meta-analyses were excluded from our analysis.

### Validity assessment

To determine the validity of the research, the quality of each case–control study was assessed by the second author according to the primary criteria for nonrandomized studies described in the Newcastle-Ottawa Scale (NOS) [14] as follows: A. selection of the study groups; B. comparability of the groups; C. ascertainment of the exposure. For cross-sectional studies, we evaluated four aspects of the quality of the studies: A. representativeness of study participants, B. proper methods to ascertain exposure, C. comparability of the analysis groups, and D. low non-response bias. Each study was scored with respect to meeting (1) or not meeting (0) each of these criteria, and a dash indicated that fulfillment of the criteria could not be determined.

### Data abstraction

All of the identified articles about suicide among Chinese subjects were examined in detail. Two reviewers independently abstracted the data from potentially relevant articles. Differences were resolved by consensus. For cross-sectional studies, sampling strategy, sample size, characteristics of participants, and information used for comparative analyses were abstracted. For case–control studies, information about the size of the case and control groups; characteristics of participants; and the number of cases/controls exposed and not exposed to the study factors were abstracted from each study. When the results of a study were published more than once, only the most complete data were included in our analysis.

### Assessment of heterogeneity

We evaluated heterogeneity between the studies using the Q test [15] and the I-squared statistic (I² = 100% x (Q-df)/Q) [16]. For the Q test, a *P* value of less than 0.10 was considered statistically significant. If *P* > 0.10, there was a lack of heterogeneity between the studies. If *P* ≤ 0.10 (i.e., heterogeneity was significant), we calculated I². If I² ≤ 50%, the heterogeneity might be acceptable; otherwise, there was significant heterogeneity between studies and we conducted a subgroup analysis. For the subgroup analyses, heterogeneity within groups was also tested.

### Data synthesis

The classification of suicide risk factors is somewhat arbitrary in current reports. To better describe and sort the risk factors, we classified them in the text as follows: demographic factors, socio-family environment, lifestyle/behaviors, negative/stressful life events and psychiatric/psychological factors. To estimate the associations between these factors and suicide, we calculated pooled odds ratios (OR) for suicide ideation, suicide attempt, and completed suicide separately, using RevMan 5.0 software (RevMan software, Version 5.0, Cochrane Collaboration, Oxford, United Kingdom). If there was no significant heterogeneity (*P* > 0.10 or *P* ≤ 0.10, but I^2^ < 50%), a fixed effects model was used to pool the data. If significant heterogeneity (*P* ≤ 0.10, I^2^ >50%) was found between studies, the meta-analysis was conducted using a random effects model. Subgroup analyses were performed to explore possible reasons for the heterogeneity.

## Results

### Description of studies

**Figure 1:**
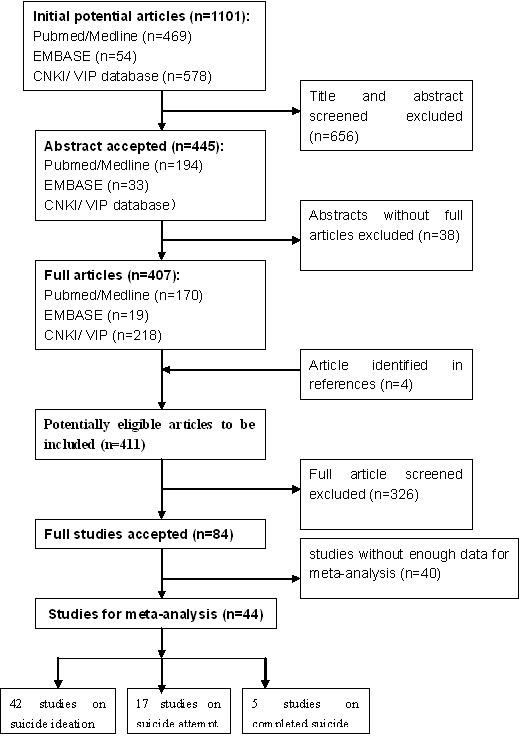
** Results of the literature search for systematic review and meta-analysis. **This figure displays the full search process used to identify studies for inclusion in this systematic review and meta-analysis.

A detailed diagram of the full review process is presented in Figure 1. We identified 1101 potentially relevant articles. After reviewing their titles, abstracts, and full text, 43 studies]. (Table 1) with 192,362 subjects had suitable data for meta-analysis and were therefore included in our study (39 cross-sectional studies and 4 case–control studies).

**Table 1:** Descriptive information for each study included in meta-analysis※

**Studies**	**Study type**	**Sampling size**	**Study population**	**Gender**	**Residence**	**Years of data collection**	**Outcome**
Zhang 1999[6]	CS	84767	16-78 years old	Female/male	Rural/urban	1989-1994	Complete suicide
Kong 2010[7]	CC	370/370	The suicides(45.30 ± 12.88)/living controls (35.03 ± 12.6)	Female/male	Rural	2005-2008	Complete suicide
Phillips 2002[8]	CC	519/536	The suicides/other injuries (>10 years)	Female/male	Rural/urban	1998-2000	Complete suicide
Li 2005[9]	CS	1653	Adolescents	Female/male	Rural/urban	1995-1999	Complete suicide
Zhang 2003[10]	CS	1421	Adolescents	Male/female	—	—	Suicide ideation
Liu 2007[12]	CS	1933	Adolescents	Male/female	Rural/urban	—	Suicide ideation
Ou 2008[13]	CS	698	College Students	Male/female	Rural/urban	2005	Suicide ideation
Ma 2003[17]	CS	853	Adolescents	Male/female	Urban	—	Suicide ideation
Fan 2008[18]	CS	2160	College Students	Male/female	Rural/urban	—	Suicide ideation
Zhang 2007[11]	CS	1294	Adolescents	Male/female	Urban	—	Suicide ideation
Zeng 2005[19]	CS	427	College Students	Male/female	Rural/urban	2003	Suicide ideation
Li 2009[20]	CY	2,012	Community members (>18 years)	Male/female	Rural/urban	2004	Suicide ideation
Yang 2008[21]	CS	8315	Adolescents	Male/female	Rural	2006	Suicide ideation, suicide attempt
Pan 2006[22]	CS	5169	Adolescents	Male/female	Rural/urban	2004	Suicide ideation
Chen 2006[23]	CS	7422	College Students	Male/female	Rural/urban	—	Suicide ideation
Yang 2007[24]	CS	3568	College Students	Male/female	Rural/urban	2006	Suicide ideation
Duan 2008[25]	CS	5,640	Community members (32–64 years)	Male/female	—	—	Suicide ideation, Suicide attempt
Hou 2010[26]	CS	239	Suicide-attempt cases(>15 years)	Male/female	—	2007-2008	Suicide ideation
Sun 2010[27]	CS	20716	Community members, >18 years	Male/female	Rural/urban	2004-2005	Suicide attempt
Bao 2009[28]	CS	1529	College Students	Male/female	Rural/urban	—	Suicide ideation
Juan 2010[29]	CS	4644	Adolescents	Male/female	Urban	2004	Suicide ideation, suicide attempt
Niu 2006[30]	CS	2,914	Patients without psychopathic problem in hospital (>15 years)	Male/female	Rural/urban	2003-2004	Suicide ideation, suicide attempt
Tian 2010[31]	CS	974	Researcher(34 ± 8)	Male/female	—	—	Suicide ideation
Sun 2008[32]	CS	943	Migrants	Male/female	—	—	Suicide ideation, suicide attempt
Li 2004[33]	CS	2032	Students in technical school	Male/female	Rural/urban	—	Suicide attempt
Yu 2004[34]	CS	1952	Adolescents	Male/female	Rural	—	Suicide ideation, suicide attempt
Xing 2005[35]	CS	4622	Adolescents	Male/female	—	2002	Suicide ideation
Gao 2001[36]	CS	2044	Adolescents	Male/female	Rural/urban	1998	Suicide ideation, suicide attempt
Zhuang 2007[37]	CS	3798	Adolescents	Male/female	urban	—	Suicide ideation, suicide attempt
Huang 2000[38]	CS	2467	Adolescents	Male/female	—	—	Suicide ideation
Zeng 2009[39]	CS	490	College Students	Male/female	Rural/urban	—	Suicide ideation
Yan 2009[40]	CS	772	College Students	Male/female	—	After 2007	Suicide ideation
Shang 2008[41]	CS	2678	College Students	Male/female	Rural/urban	2006	Suicide ideation, suicide attempt
Lin 2005[42]	CS	1100	College Students	Male/female	—	—	Suicide ideation
Zhu 2006[43]	CS	1765	College Students	Male/female	—	2004	Suicide ideation
Xu 2004[44]	CS	610	College Students	Male/female	Rural/urban	—	Suicide ideation
Zhou 2005[45]	CS	337	Adolescents	Male/female	Rural	2004	Suicide ideation
Jiang 2006[46]	CS	1514	Adolescents	Male/female	Rural/urban	—	Suicide ideation
Zhang 2009[47]	CS	15, 000	residents,	Male/female	Rural	2001	Suicide ideation
Feng 2006[48]	CS	2584	Adolescents	Male/female	urban	2004	Suicide ideation, suicide attempt
Sun 2007[49]	CS	1,021	Rural female(15–39 years)	female	Rural	—	Suicide ideation
Huan 2004[50]	CC	100/100	Suicide-attempt cases/injured by accidents	Male/female	Rural/urban	2002	Suicide attempt
Yang 2008[51]	CC	22/23	The suicide(43.1 years)/other injuries(48.2 years)	Female/male	Rural	1998-1999	Complete suicide

**※:***CC* refers to case–control study; *CS* refers to cross-sectional study.

The methodological characteristics of the included studies were then evaluated. In most of the cross-sectional studies, the participants had good representativeness. However, 7 cross-sectional studies [13,19,23,26,34,38,42] had no description of sampling methods or a small sample size. The exposure and control groups in the majority of studies were comparable, except for three studies [21,34,35] that did not compare them and seven studies [11,17,22,36-39] that did not report the non-response rate. Overall, only 3 studies [34,38,39] scored below 3 (Additional file 1: Table S1). All case–control studies scored six or above and were judged as being of high methodological quality (Additional file 1: Table S2).

### Meta-analysis

There were 42, 17, and 5 studies with dichotomous data available for a meta-analysis of the factors associated with suicide ideation, suicide attempt and completed suicide, respectively. Heterogeneity tests were carried out on all of these studies and the results are presented in Table 2.

**Table 2:** Results of meta-analysis of the studies on association between factors and suicide in Chinese population

**Factors**	**No. of studies**	**No. of participants**	**Variance between studies**		**Pooled OR**	**95% CI**	**Test for overall effect (p)**
			**Q(*p*)**	**I**^**2**^**(%)**			
**Suicide ideation**							
*Demographic characteristics*							
Gender (female)	27	71546	<0.00001	88	1.43	1.25, 1.64	**<0.00001**^**※**^
Rural residence	15	51850	<0.00001	78	0.99	0.87, 1.12	0.85^**※**^
Lower education	9	33749	<0.00001	95	1.39	0.91,2.13	0. 13^**※**^
Poor economy	8	13260	0.004	66	1.16	0.91,1.49	0.24^**※**^
*Psychiatric or psychological factors*							
Mood disorder	6	15842	<0.00001	86	5.12	3.78, 6.94	**<0.00001**^**※**^
*Socio-family environment*							
^*^Special family	8	11490	0.07	46	1.55	1.23, 1.96	**<0.00001**^**※**^
One child in family	11	23486	0.04	47	1.04	0.96, 1.13	0.33^**★**^
Poor relationship with families	2	2345	0.25	26	2.31	1.53, 3.54	**0.0001**^**★**^
Poor academic achievement	6	9354	0.44	0	1.25	1.08, 1.44	**0.003**^**★**^
Study pressure	3	12590	0.16	46	1.27	1.17,1.38	**<0.00001**^**※**^
*Lifestyle/Behaviors*							
Smoking	4	9197	0.21	34	1.67	1.45, 1.92	**<0.00001**^**★**^
Alcohol	4	10437	0.17	41	1.96	1.69, 2.27	**<0.00001**^**★**^
Drug use	3	5280	0.37	0	1.97	1.39, 2.80	**0.0001**^**★**^
*Stressful life events*							
Physical illness	9	22639	<0.00001	98	4.82	1.24, 18.68	**0.01**^**※**^
Suicide of relatives	4	14163	0.002	80	3.02	1.81,5.04	**<0. 0001**^**※**^
**Suicide attempt**							
*Demographic characteristics*							
Gender (female)	13	3462	<0.00001	79	1.09	0.80, 1.50	0.59^**※**^
rural residence	7	12006	0.004	69	0.78	0.53, 1.15	0.21^**※**^
Currently unmarried	5	24938	0.06	60	1.19	0.88, 1.60	0.27^**★**^
Lower education	2	2954	0.05	75	1.24	0.80, 1.90	0.34^**※**^
*Psychiatric or psychological factors*							
Mood disorder	4	19277	<0.00001	88	2.74	1.31, 5.74	**0.007**
*Socio-family environment*							
Poor relationship with families	4	15371	0.15	43	3.64	2.97, 4.47	**<0.00001**^**★**^
One child in family	2	3100	0.69	0	1.77	1.26, 2.49	**0.001**^**★**^
*Lifestyle/Behaviors*							
Smoking	4	7995	0.18	39	1.79	1.36, 2.35	**<0.0001**^**★**^
Alcohol use	3	7798	0.37	0	2.50	1.86, 3.36	**<0.00001**^**★**^
Drug use	2	5889	0.23	30	7.33	4.60, 11.67	**<0.00001**^**★**^
*Stressful life events*							
Poor physical health	4	8685	0.04	65	2.44	0.93, 6.41	0.07^**★**^
Suicide of relatives	3	13016	0.97	0	4.80	3.12, 7.39	**<0.00001**^**★**^
**Completed suicide**							
*Demographic characteristics*							
Gender (female)	4	397382	<0.01	73	2.35	1. 15, 4.78	**0.02**^**※**^
Lower education	4	398072	0.003	79	2.33	1.48, 3.66	**0.0003**^**※**^
Residence(rural)	3	397337	0. 04	69	2.86	1.20, 6.84	**0.02**^**※**^
Currently married	6	398710	<0.00001	96	1.14	0.50, 2.60	0.76^**※**^
*Psychiatric or psychological factors*							
Mood disorder	4	1757	0.9	0	30.54	20.46, 45.58	**<0.00001**^**★**^
*Stressful life events*							
Negative life event	3	1264	0.63	0	10.03	6.63, 15.19	**<0.00001**^**★**^
Seeking help for mood disorder	2	1215	1.00	0	5.41	3.36, 8.71	**<0.00001**^**★**^
Previous suicide attempt	2	1306	0.68	0	31.77	13.48, 74.91	**<0.00001**^**★**^
Suicide of relatives	2	1218	0.79	0	4.55	3.03, 6.85	**<0.00001**^**★**^
Pesticides stored at home	2	1543	0.60	0	1.92	1.55, 2.37	**<0.00001**^**※**^
Change of life	3	1231	0.86	0	3.79	2.56, 5.61	**<0.00001**

^*^Special family included reconstituted family, single-parent family, or non-parent family.

^**★**^Pooled data by a fixed effect model.

^**※**^Pooled data by a random effect model.

**Figure 2:**
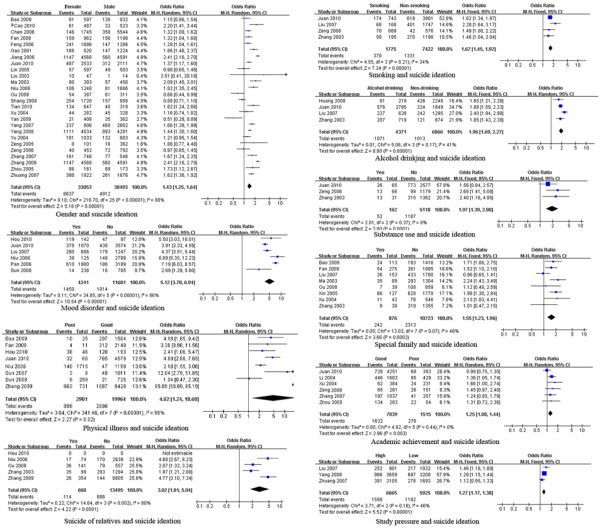
** Forest plots of factors associated with suicide ideation. **This figure shows forest plots of the meta-analysis of the association between each factor and suicide ideation. ORs and 99% CIs for each factor are given.

In terms of suicide ideation, significant heterogeneity was found for studies of the association of suicide ideation with gender, education, residence, mood disorders, physical health, suicide of relatives, and the economy. The pooled ORs and 95% CIs indicated that demographic factors (female gender), psychiatric/psychological factors (mood disorders) and negative/stressful life events (poor physical health, suicide of relative) were risk factors for suicide ideation among the Chinese population [pooled OR (95% CI): 1.43 (1.25, 1.64), 5.12 (3.78, 6.94), 4.88 (1.24, 18.68) and 3.02 (1.81, 5.04), respectively]. Lifestyle/behaviors (smoking, alcohol drinking, and substance use), family environment (single or remarried parent), poor academic achievement and study pressure were significant risk factors for suicide ideation among youth (adolescents and college students) [pooled OR (95% CI): 1.67 (1.45, 1.92), 1.96 (1.69, 2.27), 1.55 (1.23, 1.96), 1.97 (1.39, 2.80), 1.25 (1.08, 1.44) and 1.27 (1.17, 1.38), respectively] (Table 2 and Figure 2).

**Figure 3:**
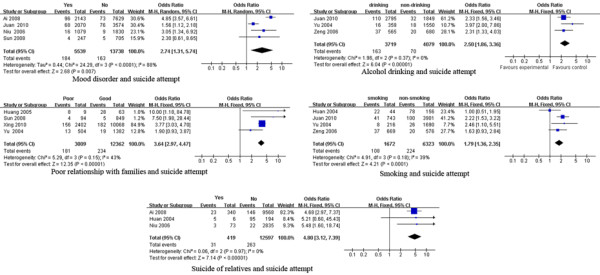
** Forest plots of factors associated with attempted suicide. **This figure shows forest plots of the meta-analysis of the association between each factor and attempted suicide. ORs and 99% CIs for each factor are given.

The factors associated with suicide attempt are shown in Table 2 and Figure 3. Heterogeneity tests identified significant heterogeneity among studies of the association of suicide attempt with gender, education, residence and mood disorders. Results of the meta-analysis showed that psychiatric/psychological factors (mood disorders), socio-family environment (poor relationship with family), lifestyle/behaviors (smoking, alcohol use) and negative life events (suicide of relatives) were significantly associated with attempted suicide [pooled OR (95% CI): 3.44 (1.93, 6.14), 3.64 (2.97, 4.47), 1.79 (1.36, 2.35), 2.50 (1.86, 3.36), 4.80 (3.12, 7.39), respectively].

**Figure 4:**
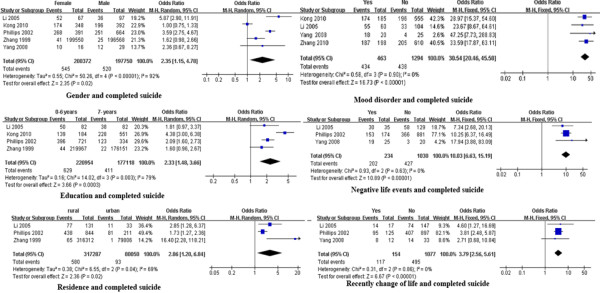
** Forest plots of factors associated with completed suicide. **This figure shows forest plots of the meta-analysis of the association between each factor and completed suicide. ORs and 99% CIs for each factor are given.

Heterogeneity tests showed that studies of the association between mood disorders, seeking help for mood disorders, previous suicide attempt, suicide of relatives, negative life events, pesticides stored at home and completed suicide lacked significant heterogeneity. According to the pooled ORs and 95% CIs, demographic factors (female gender, residence in rural area, lower education), psychiatric/psychological factors (mood disorder), negative life events and recent change in life predicted completed suicide [pooled OR (95% CI): 2.35 (1.15, 4.78), 2.86 (1.20, 6.84), 2.33 (1.48, 3.66), 30.54 (20.46, 45.58), 10.03 (6.63, 15.19), and 3.79 (2.56, 5.61), respectively](Table 2 and Figure 4).

### Subgroup analysis

**Figure 5:**
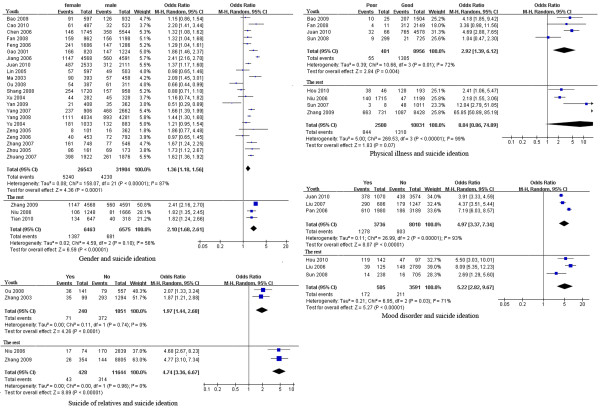
** Forest plots of subgroup analysis of factors associated with suicide ideation. **This figure shows forest plots of the subgroup analysis of the association between gender, suicide of relatives, mood disorder, and physical illness with suicide ideation by population (youth vs. the rest). ORs and 99% CIs for each factor are given.

Given the available collected data, we conducted subgroup analyses of both suicide ideation and completed suicide. Many studies have investigated the factors associated with suicide ideation among youth (adolescents and college students). We carried out subgroup analyses of the association of suicide ideation with gender, mood disorders, physical health, and suicide of relatives by population, namely, youth vs. the rest of the population. The heterogeneity test showed that studies of the effects of physical illness [13,28,39] and suicide of relatives [9,11] on youth suicide ideation lacked heterogeneity, but studies on the effect of gender and mood disorder had significant heterogeneity (*P* < 0.00001). For factors associated with suicide ideation in the rest of the population, studies lacked heterogeneity, except for those of physical illness (*P* < 0.00001) [28-32]. The pooled ORs (and 95% CIs) showed that female gender, mood disorders, physical illness, and suicide of relatives were still risk factors for youth suicide ideation [pooled OR (95% CI): 1.36 (1.18, 1.56), 4.97 (3.37, 7.34), 2.92 (1.39, 6.12) and 1.97 (1.44, 2.68), respectively] (Figure 5).

**Figure 6:**
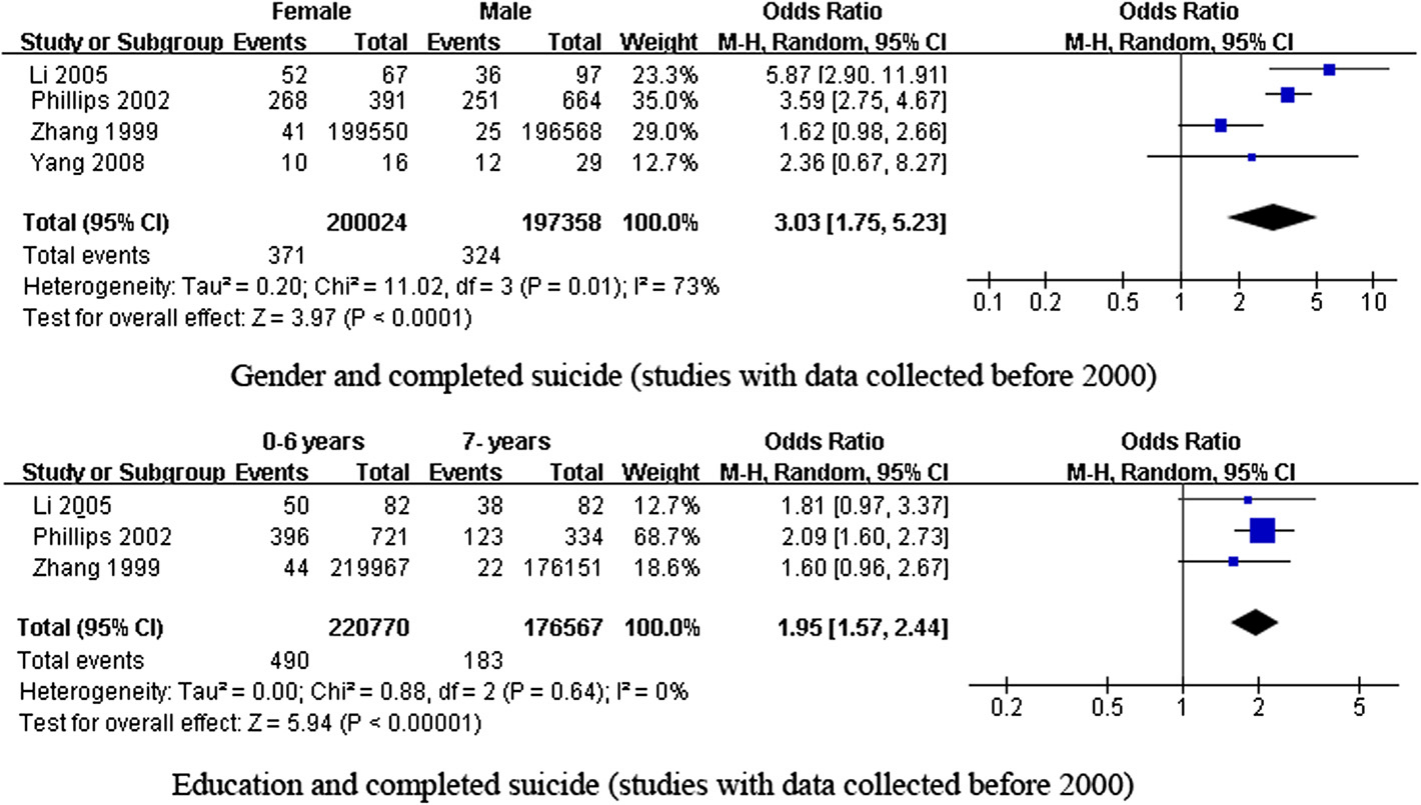
** Forest plots of subgroup analysis of factors associated with completed suicide. **This figure shows forest plots of the subgroup analysis of the association between gender and education with completed suicide by the period of data collection (before 2000 vs. 2000 and after). ORs and 99% CIs for each factor are given.

For the factors associated with completed suicide, the subgroup analyses were performed by year of data collection. Regarding gender and completed suicide, when we excluded study data collected after 2000 [6], the studies using data collected before 2000 still had significant heterogeneity. The pooled results indicated that females had an even higher risk of completed suicide than males [pooled OR (95% CI): 3.03 (1.75, 5.23)]. As for education and completed suicide, when we excluded study data collected after 2000 [7], the studies [7,8] using data collected before 2000 lacked heterogeneity. The pooled OR still indicated that lower education was a risk factor for completed suicide in China [pooled OR (95% CI): 1.95 (1.57, 2.44)] (Figure 6).

## Discussion

In recent years, much attention has been given to suicide prevention in China. Since 1999, suicide prevention has been listed as a mental health priority of the Ministry of Health in China [33]. We systematically reviewed a range of risk factors of suicidal behaviors in China. Given that there is a large body of literature on the associations between numerous factors and suicidal behaviors, our meta-analysis only provides a review of the strongest and most consistently reported factors.

In Western countries, males have a higher risk of completed suicide than females [52]. In China, based on the studies included in our meta-analysis, the opposite pattern was identified; females had a 2.35-fold higher risk of completed suicide than males. In the subgroup analysis, when we excluded the study [7] with data covering 2005–2008, the pooled results of studies with pre-2000 data indicated that women had an even higher risk (3.03-fold) of suicide, which hinted that the impact of gender on suicide in China is gradually decreasing. This result is consistent with that of [Zhang et al. 5], who found that the female suicide rate decreased more than the male suicide rate during the period between 1987 and −2008 (20.4 to 6.2/100,000 vs. 14.9 to 7.0/100,000, respectively) [5],which is due to the economic development over the past decades resulting in increase of female social status in China, particularly in rural areas [5]. Our meta-analysis indicated that people living in rural areas and those with less education have a higher risk of completed suicide (pooled OR = 2.86 and 2.33, respectively). These results may explain the high suicide rates in rural areas of China [5].

Socio-family environmental factors, such as personal relationships, family violence, social and family conflicts and a sense of isolation, were significantly associated with a higher risk of completed suicide [52-56]. Previous reviews also report that multiple social and interpersonal factors, such as parent/family and peer relationships, are important for our understanding of adolescent suicidality [57]. The present meta-analysis also indicated that being in a special family (single or remarried parent), poor academic achievement and study pressure increase the risk of developing suicide ideation among adolescents and college students (pooled OR = 1.50, 1.25 and 1.27, respectively). These factors may influence the development of personality or psychological disorders among youth and adolescents [58]. Our results highlight the importance of establishing harmonious family and social environments for suicide prevention. School factors should also be considered for student suicide prevention.

As for the psychiatric/psychological factors, it has been reported that mental disorders, mood disorders (severe depression, anxiety, sadness, and so on), and personality disorders are major risk factors for suicidality worldwide, particularly in Western countries [52,55,57,59-61]. However, Zhang et al. pointed out that in China, although still important, psychiatric factors are not the main factors associated with suicide [5]. Our meta-analysis similarly found that people with a mood disorder had 2.48-, 3.44- and 20.31-fold higher risk of suicide ideation, suicide attempt and completed suicide in China, respectively. However, the included studies qualitatively described psychiatric/psychological factors using with/without sadness, depression, and despair, rather than quantitatively measured it with a standard instruments, which may result in different criteria implemented in different studies to decide with or without these mood disorders. Therefore, a standard scale in the future studies is needed for measuring mood disorders in order to compare between studies.

Negative/stressful life events influence health in many respects. Previous studies have reported that stressful life events are associated with suicide. For example, poor physical health, disabilities, history of suicide attempts, life events, and suicide of others are significant suicide risk factors [54,57,61-63]. An earlier study also reported that the presence of legal/disciplinary problems, potentially fatal illnesses, persistent stress, previous self-harm, and family history of suicide are risk factors for suicidal behaviors [52,55,64,65]. Our meta-analysis indicated that people who had experienced a negative/stressful life event or a recent change in life, and those who had previously attempted suicide or had relatives/friends who had committed suicide had a higher risk of completed suicide. We also found that poor physical health and exposure to the suicide of a family member were risk factors for suicide ideation and suicide attempt. Indeed, a negative life event can trigger suicide or suicidal behavior because negative life events are associated with psychological disorders [66,67].

Earlier studies have identified alcohol, smoking and drug disorders as being suicide risk factors [37,68-70]. Misuse of alcohol or drugs was also a risk factor for suicide ideation and suicide attempt [55]. Our meta-analysis similarly found that among youth, smoking, alcohol use, and drug use were risk factors for suicide ideation (pooled OR = 1.67, 1.96 and 1.97, respectively), and smoking and alcohol use were risk factors for suicide attempt (pooled OR = 2.03 and 2.50, respectively). It has long been recognized that substance abuse (alcohol and drug use) is frequently correlated with psychiatric disorders [71,72], and that smoking in youth is associated with psychiatric disorders (depression and anxiety) [73,74], which may explain how these unhealthy lifestyle behaviors increased the risk of suicidal behaviors.

Our meta-analysis also indicated that being an only child, pesticides being stored at home, a poor economy, lower social support, and seeking psychological help in hospitals were risk factors for suicide in China. However, further study is needed to confirm these risk factors because there were only a few studies of these factors included in our meta-analysis, all with small sample sizes.

This review provides a relatively comprehensive picture of risk factors for suicidality in China. However, our meta-analysis has some limitations. First, biases have been introduced because non-published data, papers without full text and papers published in languages other than English or Chinese were not included. Second, there was a lack of uniform measurement of complex socio-psycho-behavioral factors among the included studies, and they covered widely different population groups, producing heterogeneity between the studies even with a subgroup analysis. Third, it is possible that the studies included in this review underestimated the association between the risk factors and suicide behaviors. Participants of studies on sensitive topics such as suicide often underreport behavior to avoid embarrassment or possible stigma [75]. Fourth, the vast majority of the included studies were cross-sectional and did not allow for inferences on cause-effect mechanisms.

Our study identified four implications for the future. First, to draw comparisons between studies of different populations and regions, it is important to emphasize the use of standardized measures of socio-psychological factors and to place adequate focus on very specific population groups in any future studies. Second, risk assessment is more necessary to identify modifiable or treatable high-risk factors and available protective factors in order to take effective countermeasures Third, most of the present studies were cross-sectional studies, therefore the future researches designed with case–control or cohort studies are needed in order to make reliable inferences on cause-effect mechanisms. Fourth, there is an urgent need to emphasize further study of interventions to prevent suicide. Intervention programs in China that simultaneously address multiple factors associated with suicide would be most appropriate since suicide is multi-factorial health problem [76].

## Conclusions

In conclusion, suicide risk factors in China are very complex. People in rural areas have a higher risk of developing suicide ideation and committing suicide. Socio-family environment, psychiatric/psychological factors, lifestyle, and stressful life events are the main risk factors for suicidal behavior in China. Notably, the impact of gender on suicide is gradually decreasing. For youth, interventions should focus on changing family environments, school factors and unhealthy lifestyles.

## Competing interests

The authors declare that they have no competing interests.

## Authors’ contributions

JC designed the study and directed its implementation, including quality assurance and control. YiL and YaL collected, analyzed and interpreted the data; YiL draft the manuscript, JC and YaL polished the draft. All authors approved the final version for submission to BMC Public Health.

## Pre-publication history

The pre-publication history for this paper can be accessed here:

http://www.biomedcentral.com/1471-2458/12/524/prepub

## Supplementary Material

Additional file 1 **Table S1. **Quality assessment of cross-sectional studies, and **Table S2. **Quality assessment of case-control studies. Click here for file
