# Supplementary material for: Factors associated with suicidal behaviors in mainland China: a meta-analysis
Source: BMC Public Health. 2012 Jul 16;12:524. doi: 10.1186/1471-2458-12-524 (PMC3490836; doi:10.1186/1471-2458-12-524)
Supplement: Additional file 1 — Table S1. Quality assessment of cross-sectional studies, and Table S2. Quality assessment of case-control studies. [file 1471-2458-12-524-S1.doc]

**Supplement table1 Quality assessment of cross-sectional studies※**

| Studies | Study design | A | B | C | D | Total score |
| --- | --- | --- | --- | --- | --- | --- |
| Zhang 1999[6] | Cross-sectional | 1 | 1 | 0 | 0 | 2 |
| Li 2005[9] | Cross-sectional | 1 | 1 | 1 | 1 | 4 |
| Zhang 2003[10] | Cross-sectional | 1 | 1 | 1 | 1 | 4 |
| Liu 2007[11] | Cross-sectional | 1 | 1 | 1 | 1 | 4 |
| Ou 2008[12] | Cross-sectional | 1 | 1 | 1 | 1 | 4 |
| Ma 2003[13] | Cross-sectional | 0 | 1 | 1 | 1 | 4 |
| Fan 2008[17] | Cross-sectional | 1 | 1 | 1 | 1 | 4 |
| Zhang 2007[18] | Cross-sectional | 1 | 1 | 0 | 1 | 3 |
| Zeng 2005[19] | Cross-sectional | 0 | 1 | 0 | 1 | 2 |
| Li 2009[20] | Cross-sectional | 1 | 1 | 0 | 1 | 3 |
| Yang 2008[21] | Cross-sectional | 1 | 1 | 1 | 0 | 3 |
| Pan 2006[22] | Cross-sectional | 1 | 1 | 1 | 0 | 3 |
| Chen 2006[23] | Cross-sectional | 0 | 1 | 1 | 0 | 2 |
| Yang 2007[24] | Cross-sectional | 1 | 1 | 1 | 0 | 3 |
| Duan 2008[25] | Cross-sectional | 1 | 1 | 1 | 0 | 3 |
| Hou 2010[26] | Cross-sectional | 0 | 1 | 1 | 0 | 2 |
| Sun 2010[27] | Cross-sectional | 1 | 1 | 1 | 0 | 3 |
| Bao 2009[28] | Cross-sectional | 1 | 1 | 1 | 1 | 4 |
| Juan 2010[29] | Cross-sectional | 1 | 1 | 1 | 1 | 4 |
| Niu 2006[30] | Cross-sectional | 1 | 1 | 1 | 1 | 4 |
| Tian[2010][31] | Cross-sectional | 1 | 1 | 1 | 1 | 4 |
| Sun 2008[32] | Cross-sectional | 1 | 1 | 1 | 1 | 4 |
| Li 2004[33] | Cross-sectional | 1 | 1 | 1 | 1 | 4 |
| Yu 2004[34] | Cross-sectional | 0 | 1 | 1 | 1 | 3 |
| Xin 2005[35] | Cross-sectional | 1 | 1 | 1 | 1 | 4 |
| Gao 2001[36] | Cross-sectional | 1 | 1 | 1 | 1 | 4 |
| Zhuang 2007[37] | Cross-sectional | 1 | 1 | 1 | 1 | 4 |
| Huang 2000[38] | Cross-sectional | 0 | 1 | 1 | 1 | 3 |
| Zeng 2009[39] | Cross-sectional | 1 | 1 | 1 | 1 | 4 |
| Yan 2009[40] | Cross-sectional | 1 | 1 | 1 | 1 | 4 |
| Shang 2008[41] | Cross-sectional | 1 | 1 | 1 | 1 | 4 |
| Lin 2005[42] | Cross-sectional | 0 | 1 | 1 | 1 | 3 |
| Zhu 2006[43] | Cross-sectional | 1 | 1 | 1 | 1 | 4 |
| Xu 2004[44] | Cross-sectional | 1 | 1 | 1 | 1 | 4 |
| Zhou 2005[45] | Cross-sectional | 1 | 1 | 1 | 1 | 4 |
| Jiang 2006[46] | Cross-sectional | 1 | 1 | 1 | 1 | 4 |
| Zhang 2009[47] | Cross-Sectional | 1 | 1 | 1 | 1 | 4 |
| Feng 2006[48] | Cross-sectional | 1 | 1 | 1 | 1 | 4 |
| Sun 2007[49] | Cross-sectional | 1 | 1 | 1 | 1 | 4 |

※ A. representativeness of study participants, B. proper methods to ascertain exposure, C. comparability of comparing analysis groups and D. lower non-response bias

**Supplement table 2 Quality assessment of case-control studies＊**

| Studies | Study design | A | | | | B | | C | | | Total score |
| --- | --- | --- | --- | --- | --- | --- | --- | --- | --- | --- | --- |
|  |  | A1 | A2 | A3 | A4 | B1 | B2 | C1 | C2 | C3 |  |
| Kong 2010[7] | Case-control | 1 | 1 | 1 | 1 | 1 | 1 | 1 | 0 | 1 | 8 |
| Phillips 2002[8] | Case-control | 1 | 1 | 0 | 1 | 1 | 1 | 1 | 0 | 1 | 7 |
| Huan 2004[50] | Case-control | 1 | 1 | 1 | 1 | 1 | 0 | 1 | 1 | 1 | 8 |
| Yang 2008[51] | Case-control | 0 | 1 | 0 | 1 | 1 | 1 | 1 | 1 | 0 | 6 |

＊A. selection of the study groups (A1 right case definition, A2 right controls definition, A3 the representativeness of the cases, A4 the representativeness of controls); B. comparability of the groups (B1 control of main confounders, B2 control of any additional factor); C. ascertainment of the exposure (C1 appropriate method of exposure ascertainment, C2 same method of exposure ascertainment for cases and controls, C3 same non-response rate of case and control groups).
